# The science behind the wool industry. The importance and value of wool production from sheep

**DOI:** 10.1093/af/vfab005

**Published:** 2021-05-17

**Authors:** Emma K Doyle, James W V Preston, Bruce A McGregor, Phil I Hynd

**Affiliations:** 1 School of Environmental and Rural Science, University of New England, Armidale, NSW 2551, Australia; 2 Rare Fibre, Melbourne, VIC 3056, Australia; 3 School of Animal and Veterinary Sciences, The University of Adelaide, Adelaide, SA 5001, Australia

**Keywords:** sheep, skin health, wool, wool processing, wool quality

**Implications**

- This paper outlines the wool industry and highlights wool as a textile fibre. The wool industry has optimized the production of a niche product that has eco-positioned itself due to its inherent natural properties of being a natural, biodegradable product that offers consumer comfort and health benefits.
- Research into the breeding and management of sheep on-farm, has developed a raw product that is easier to process or has superior woolen attributes.
- Skin follicle formation and subsidiary glands affect wool production and quality. By understanding how wool follicle cells initiate and develop, producers are able to improve fibre quality.

## Introduction to Wool Production and Demand

Sheep and wool production occurs in a number of areas of the world. The production method, however, has been considered somewhat diverse. Wool production can collectively be the production of keratin fibers from a range of animals. This can include the production of cashmere, alpaca, mohair, angora, yak, elk, and camel fiber. Fiber characteristics from sheep wool can vary depending on the sheep breed, its age, the environmental grazing conditions, local market requirements, and export opportunities for the country of origin.

On an international scale, wool production is a small trade (Textile Exchange, 2019). The inception of manmade fibers in the 1880s has seen considerable shrinkage in the textile market share of wool. Wool production represents about 1% of the global supply of textile fibers (Table 1). Apparel wool from sheep contributes about half of that amount. The contribution of wool has fallen by about half over the past 20 yr as wool production has declined, and the production of manmade fibers has nearly doubled (IWTO, 2019).

**Table 1.** World supply of textile fibers

Fiber	Million tonnes	Percentage of market
Polyester	55.1	51.5
Cotton	26.1	24.4
Cellulosic	6.7	6.2
Other plant fibers, including flax, hemp, jute, and coir	6.1	5.7
Polyamide	5.4	5.0
Other manmade	6.1	5.7
Wool sheep	1.1	1.0
Wool other animals	0.05	
Silk	0.16	0.1
Feathers, down	0.32	0.3
Total	**107**	99.9%*

Source: Textile Exchange (2019).

*The total may not be 100% due to rounding errors.

Similarly, as wool production has decreased, there has been a reduction in demand for woolen fabrics in the last two decades (IWTO, 2019). Traditionally, apparel wool was used either as outer knitwear or as woven suiting attire. Research indicates that there is a trend away from these markets due to:

- Increasing casualization of the workforce;
- Limited trans-seasonal clothing options;
- Attitudes on discretionary spending during unfavorable economic conditions.

### Casualization of workforce

Data show a consistent decreasing demand trend for woven suiting fabrics (IWTO, 2019). This is consistent with trend of the casualization of work wear and the importance of comfort and loungewear. This has been further compounded by recent requirements for employees to work from home due to recent COVID-19 pandemic restrictions.

### Limited trans-seasonal clothing options

Traditional markets rely heavily on the autumn–winter months of countries in the northern hemisphere. Consumers in this environment provide demand based on the requirement of needing warmth from knitted outerwear. The high degree of seasonality to this market limits sales throughout the warmer months of the year (Cottle, 2010). Additionally, workplaces are now heated.

### Attitudes on discretionary spending during unfavorable economic conditions

The woven suiting industry and to a lesser extent outer knitwear are heavily reliant on positive economic conditions where wool consumption is related to the consumer’s “ability to pay rather than willingness to pay” (Rowe, 2010). As textile spending is classified as discretionary spending, there is generally a trend away from textile trading during tough economic conditions.

### Future demand for wool

Future demand for wool will be determined by its ability to capitalize on new emerging markets. Due to diminishing returns in traditional markets, new markets such as the next-to-skin knitwear market (Rowe, 2010) offer an area of growth for wool. Wool marketing has focused on extending the use of wool into nontraditional markets. This includes the next-to-skin knitwear and athleisure market. These markets require wool to be worn as a base layer or as described as “next to skin”, which requires the fiber to have low fiber diameter (less than 18 μm) and capitalize on the unique fiber characteristics, such as breathability, resisting odor, and moisture-wicking capabilities. Additionally, the clean, green eco-positioning of wool according to the use of the life cycle assessment to quantify its sustainability position makes it attractive to the environmentally savvy consumer. The next-to-skin knitwear market does require certain specifications to suit this market. Wool must be soft to touch, also known as the handle of the fabric, and absent of considerable prickle predominately caused by coarse fibers (over 30 μm) to develop a level of consumer comfort (Naebe et al., 2015). Australia typically producers 95% of the world’s wool production that is finer than 19.6 µm (Cottle, 2010).

### Limitations to the expansion of wool as a textile fabric

Wool is approximately four to seven times more expensive to produce and process compared with manmade fibers and other natural fibers such as cotton (Cottle, 2010). Naturally, to recover this cost, the selling price point of wool textiles needs to be significantly higher; therefore, wool needs to be marketed as a luxury niche product. Marketing has targeted the rising middle-class Asian consumers to purchase luxury wool items.

### Economics of fiber production

The commercial significance of the physical properties of raw wool is summarized in Table 2. Mean fiber diameter is by far the most important physical property affecting processing performance, fabric properties, consumer evaluation, and price per kilogram. Some physical properties are of great importance in early and/or later stage processing, whereas others have lesser importance depending on the defined end use for which the fiber is destined. These physical properties directly affect the speed of processing, processing yield, quantity of waste products, yarn quality, dyeing performance, visual attributes, handle attributes, fabric properties, cost of product, and appeal to customer. Cottle and Baxter (2015) reviewed the testing requirements for important physical properties of wool.

**Table 2.** The importance of wool attributes to wool processing

Characteristics	Processing significance	Importance to scouring and topmaking	Importance to yarn and cloth manufacturing
Mean fiber diameter	Affects hauteur, spinning limits for yarn, fabric mass per unit area, fabric prickliness, and softness	****	****
Length	Major contributor to hauteur and yarn quality	***	***
Washing yield	Measures quantity of clean fiber	****	
Vegetable matter amount and type	Impacts carding and combing yield and contributes to hauteur and fabric quality	***	**
Strength	Major contributor to hauteur	***	
Crimp (fiber curvature)	Affects hauteur, yarn evenness, fabric properties, and handle	**	**
Clean fiber color	Affects dying ability		*
Suint/moisture content	Affects wool color	*	*
Handle	Affects softness of fabrics	*	**
Weathering	Affects hauteur and dying ability	*	*

Source adapted from Anon (1973), Aitken et al. (1994), and Cottle (2010). Hauteur is defined as fiber length after early stage processing.

****Most important; ***major; **secondary; and *minor.

Research has expanded the understanding of the effects of wool crimp (fiber curvature) on processing, knitted fabric properties, and wearer comfort (McGregor and Postle, 2009; McGregor et al., 2015a). The importance and influence of wool handle have been recently reviewed by Preston et al. (2016). The challenge for producers is to produce wool acceptable to the wool value chain in a variable environment. Inconsistent rainfall is a challenge to wool producers as pasture growth is limited by rainfall. Inconsistent pasture growth will lead to a decline in wool quality traits, such as staple strength, which is important for early stage processing. There are many on-farm factors that affect the physical properties of wool, including nutrition, reproduction, health, and management, and they are reviewed elsewhere (McGregor et al., 2016).

### Influence of shearing on sheep management, wool production, and wool quality

In a sheep enterprise, lambing and shearing are two important husbandry practices that a producer can alter the timing of to improve productivity. The shearing event induces a cold response, which can result in an increased feed consumption and consequently metabolic rate. There is renewed interest in the implications of shearing to increase metabolic response, the benefits of an increased condition score post shearing, and thus improvements in reproduction rates from a strategic shearing event. An increased fertility rate potentially allows the producer to increase the reproduction rate and thus profitability.

The wool quality and production, such as staple strength, length, and fleece weights, are manipulated by the timing of the shearing event (McGuirk et al., 1966). Dust penetration will also affect the wool yield percentage and profitability of the wool production. Sheep with long wool during dusty summer conditions will have a higher dust penetration and increased contaminants in the wool. Dust penetration is also highly correlated with wool staple weathering (degradation by environment), which increases noil (waste or short fiber) losses during early stage processing and affects dying potential (Holt et al., 1994). Practices such as visually selecting for wool with additional wool grease content have shown to reduce the level of dust penetration along the staple length.

## Fiber Production in the Skin

The development of the modern wool-producing sheep is a triumph of genetics and breeding over the past 200 yr from “primitive” sheep characterized by: fibers that were coarse (>30 to 120 µm); variably pigmented; highly variable in diameter and length (typically the animals had effectively two coats: an outer coarse coat and an inner finer coat); typically long, crimpless fibers; fibers that shed on a regular seasonal basis; and fibers that were medullated (contained an air core). The density of the fibers in the skin and the total fleece weights of such sheep werelow. Indeed, this description defines “hair” in contrast to “wool,” which is characterized by: fine and ultrafine fibers (typically between 10 and 20 µm), high follicle density in the skin, high clean fleece weights, uniform fiber length and diameter (low coefficients of variation in both), high crimp frequency, regular crimp frequency, very white fibers, and almost continuous fiber production with little or no seasonal shedding. This transformation reflects strong selection pressure on the desired traits, many of which were apparent in the Merino genotypes in Saxony and Spain.

**Figure 1.**
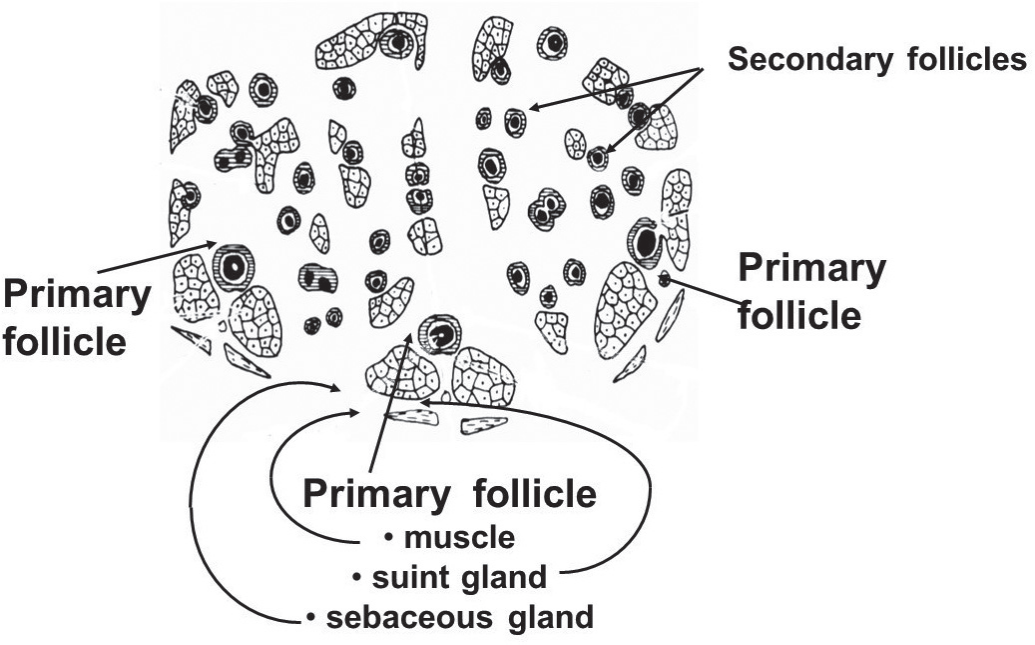
Transverse section of sheep skin (Merino) showing the typical pattern of three primary follicles (with associated sweat or suint glands and large sebaceous glands) and secondary follicles (with sebaceous glands only).

**Figure 2.**
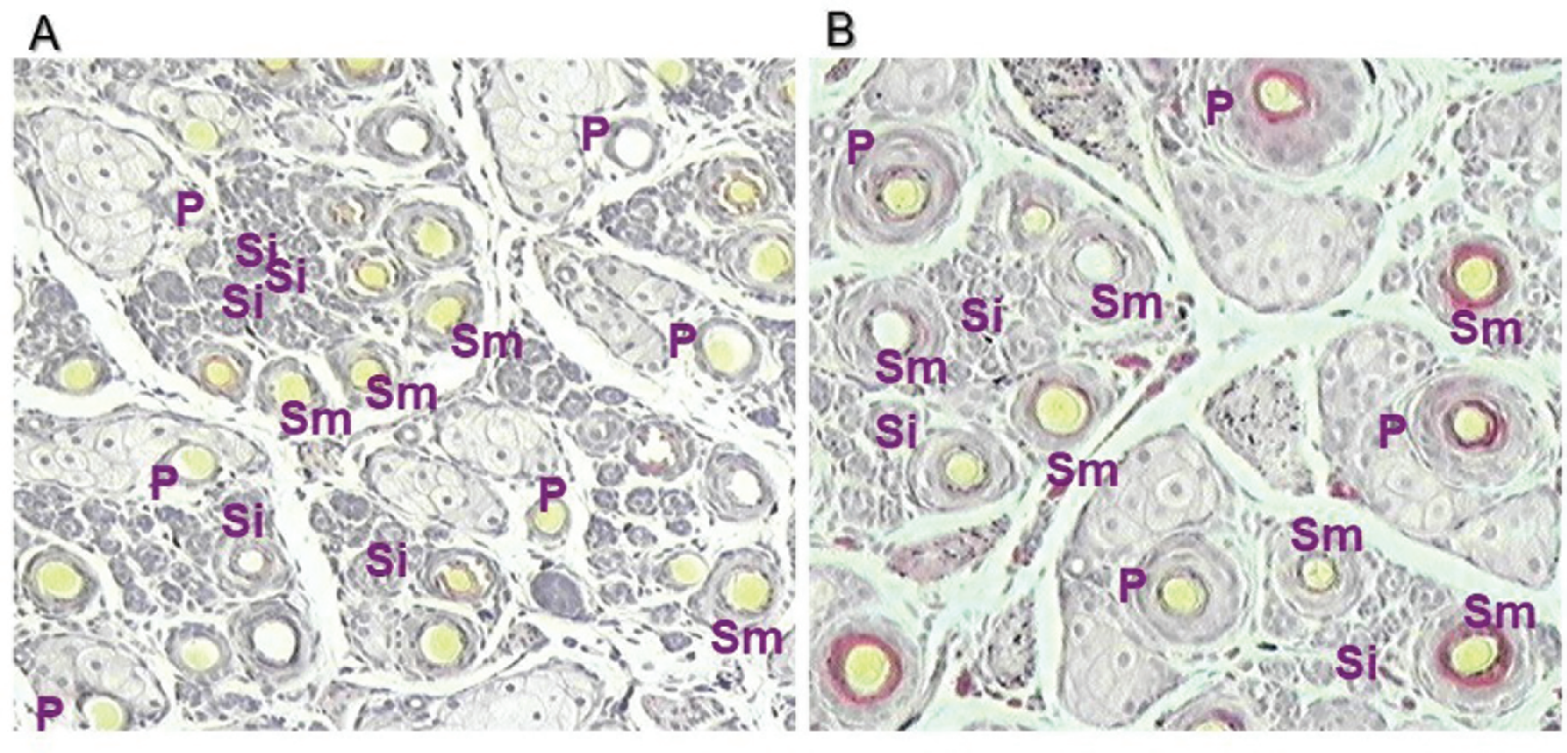
Transverse section of the skin of a Merino sheep at day 135 of gestation (A) and at birth (B) showing primary follicles (P), immature secondary follicles (Si), and mature secondary follicles (Sm). Immature secondary follicles are not yet producing a fully formed fiber.

All of the economically important traits of wool including clean fleece weight, mean fiber diameter, fiber diameter variability, fiber length, wool “style” (crimp frequency, crimp definition, crimp regularity, wool color, and dust penetration), and staple strength are determined largely by the characteristics of the follicle population, which is initiated in the skin during fetal development. Follicles are initiated in the skin in “waves” (Hardy and Lyne, 1956). The first wave is the formation of primary follicles (from days 65 to 100 of gestation); the second wave is the initiation of secondary original follicles (days 90 to 130 of gestation); and the third is the branching of these secondary follicles, known as secondary-derived or branched secondary follicles (from days 100 to 130 of gestation) From this point, there is no further initiation of secondary wool follicles. However, the initiated wool follicles will continue to mature up to approximately 4 wk post birth. Primary follicles are characterized by the presence of sweat glands (sudoriferous glands) and an arrector pili muscle. All follicle types have associated sebaceous glands, which deposit the wool grease or lanolin onto the fiber during fiber production. (Figures 1 and 2).

The skin follicle population is complete by about 4 mo of age after birth. After this age, follicle density declines as the surface area of the sheep is related allometrically to the (live weight)^0.67^ of the sheep. Consequently, it has been shown that the mean fiber diameter of Merino sheep is proportional to the cube root of animal size (live weight)^0.33^. This is in accord with the allocation of nutrients to follicle cross-sectional area being proportional to the increase in skin surface area arising from changes in the size of the animal. Thus, fiber diameter changes in a “proportionate” manner to the size of the animal (McGregor and Butler, 2016).

### Production of wool fibers in a follicle

The wool follicle has three major regions of fiber production: the follicle bulb or germinative region, the zone of keratinization, and the zone of final hardening. Cells multiply rapidly in the follicle bulb and the daughter cells or transient amplifying cells migrate distally up the follicle. During this migration, they are rapidly synthesizing keratin, the high-sulfur amino acid fiber protein. Keratin is synthesized from amino acids derived from the surrounding blood vessels and delivered to the cells by amino acid transport systems (Thomas et al., 2007). Wool proteins are then formed by the normal gene transcription/translation mechanisms of mammalian cells (Fratini et al., 1994). The inner root sheath, which surrounds the fiber cells, is also produced by the cells produced in the follicle bulb. In fact, most of the cells produced in the bulb produce this sheath and not the actual fiber (Hynd, 1989). The root sheath hardens before the fiber cells and produces a “dye” through which the wool cells are cast and shaped. As the cells approach the end of the keratinization zone, the sheath cells are resorbed and the fiber cells dehydrate and hardens. The hardening is a result of the production of disulfide bonds between sulfur atoms on the cysteine residues in the keratin protein.

Wool fibers contain two major cell types: the cortical cells, which form the bulk of the fiber and which are thin, elongated cells (approximately 5 µm wide and 100 µm long) (Hynd, 1989), and the cuticle cells, which are thin (1 µm) flat cells that surround the fiber and overlap with each other to produce the typical scale pattern seen on animal fibers (Meyer et al., 2002). The cortical cells are of two types: the paracortical cells, which are typically high in sulfur-containing amino acids, and the orthocortical cells, which are characterized by lower-sulfur proteins (Fratini et al., 1994). Wool fibers are characterized by regular repetitions of crimping or curling of the fiber. The higher the frequency of crimping, generally the finer the diameter of the fiber, although the relationship is far from perfect. The crimp in wool is a result of a combination of differential hardening of cells on one side of the fiber relative to the other, which is associated with differential rates of cell production on either side of the dermal papilla, the small tongue of tissue that invaginates the follicle bulb (Hynd et al., 2009).

### Effects of genetics and nutrition on wool growth and quality

The effects of genotype and nutrition on wool growth and wool quality are evident through changes in the skin follicle population described above and through the rates of cell production, keratinization, and elongation. From a genetic viewpoint, the main trait affecting wool “quality” and quantity is follicle density. Generally, sheep with high mean fiber diameter have higher clean fleece weights but there are significant genetic deviations from unity allowing producers to select animals that have genetically not only low mean fiber diameter but also high clean fleece weight. The linking trait is follicle density; high follicle density is associated with both low mean fiber diameter and high fleece weight. Higher follicle density is associated with both lower fiber diameter, but lower fiber diameter is associated with lower clean fleece weight on average. Importantly, however, the relationship between mean fiber diameter and clean fleece weight is highly variable and that means there is substantial opportunity to identify genotypes of sheep, which have not only a low fiber diameter but also a relatively high clean fleece weight. These animals have more total wool follicles because of higher follicle density (number per unit area of skin) and greater total skin area (because the animals are larger and may have greater skin surface area/body weight). The latter is due to greater wrinkling of the skin (i.e., skinfold), which can be detrimental for other reasons (blowfly attractiveness and difficulty shearing). Again, however, high fleece weight and low fiber diameter are achievable without increasing wrinkle because the relationship between wrinkle and those traits are genetically highly variable.

**Figure 3.**
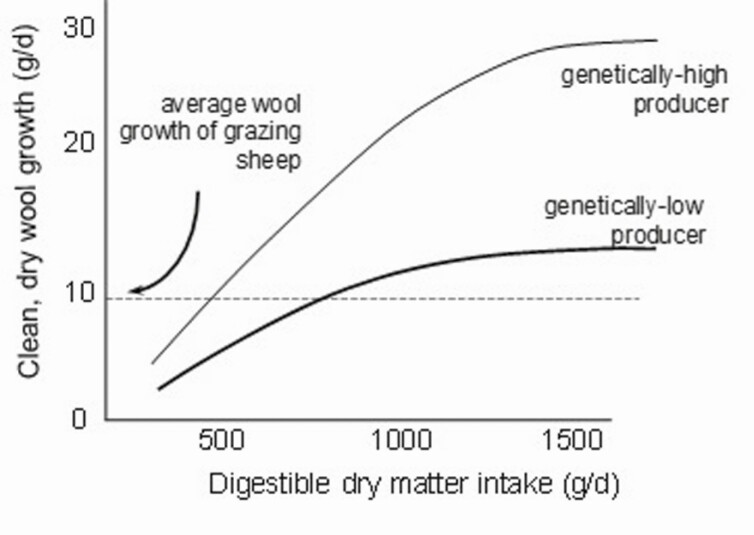
Relationship between digestible dry matter intake (g/day) and clean wool growth rate for sheep with a high or low genetic propensity for wool growth rate (after Hynd, 2019).

Nutrition has strong effects on the rate of wool production and most of the quality traits. Nutrition during pregnancy influences the initiation of follicles in the skin of the fetus as described earlier. Poor ewe nutrition during follicle initiation reduces the number of follicles initiated, with permanent negative effects on lifetime wool production (reduced fleece weights) and fiber diameter (increased diameter) (Schinckel and Short, 1961). Postnatal nutrition affects wool growth as shown in Figure 3.

The rate-limiting component of the feed for wool growth is protein and specifically the sulfur-containing amino acids cyst(e)ine and methionine (Reis and Schinckel, 1963). In general, however, on most of the feeds, the supply of limiting amino acids is reflected best by the total dry matter intake and hence the strong relationship as shown in Figure 3. Note, the nature of the relationship between feed intake and wool growth is one of diminishing returns, which means that the “efficiency” of wool growth declines as the intake increases. The rate of this decline depends on the genotype of the animal such that genetically high-producing sheep are more efficient at all intake levels but particularly at high intake rates. This has important implications for stock management in that sheep at high stocking rates will produce more wool per hectare than sheep at low stocking rates even if all feed on offer is consumed under both scenarios. Importantly too, the sheep at higher stocking rates will produce fleeces that are lower in mean fiber diameter, which has additional economic benefits.

## Wool Characteristics and Processing

The greasy wool physical properties present at shearing have a direct impact on the processing performance of wool into yarn and fabrics. Table 2 describes the wool characteristics that will have an impact on yarn quality.

### Fiber diameter

Fiber diameter describes the mean diameter of the wool fiber, measured in micrometers (µm) of the greasy wool, and impacts on the yarn thickness and fabric mass per unit area (Martindale, 1945). The fiber diameter is the most important wool trait, which affects the price of greasy and clean wool and will impact on the processing performance (the finer the wool fiber the more expensive, but slower the processing), level of entanglement during the scouring process (finer wools have higher entanglement), and fiber breakage (finer wools have higher breakage). Fiber diameter can influence fiber length (hauteur) and also the amount of wool waste (Romaine) during topmaking (AWTA, 2004). The wool fabric wearer comfort and prickle response are directly correlated with fiber diameter (McGregor et al., 2013) as well as fabric handle (McGregor et al., 2015b; Preston et al., 2016).

### Staple length and strength

Staple length and staple strength will be discussed here, due to both impacting early stage processing. Staple length and staple strength are measured using the ATLAS (Automatic Tester of Length and Strength) machine. Staple length is a measure of the length of the wool staple (measured in millimeters). Staple length is important in predicting hauteur, which is an estimate of fiber length after topmaking (AWTA, 2004). Staple length is, therefore, measured prior to sale in its greasy raw wool form. Discounts are applied to short and long staple length (approximately <60 and >100 mm). More recently, it is not uncommon for producers to manage staple length by shearing at intervals less than 12 mo. As previously mentioned, there can be additional sheep production benefits with this.

Staple strength is the measure of the force (Newtons) required to break a given thickness of wool staple or bundle (ktex). Staple strength will estimate the level of fiber breakage during topmaking, which will influence hauteur. Wool that is weak or “tender” is discounted (<32 N/ktex) and expected not to be able to sustain the rigors of carding and combing processes and produce a higher level of Romaine (wool loss at combing; AWTA, 2004). The position of break of the wool staple is important, as breaks in the middle of the staple will result in shorter hauteur, which will affect potential end use.

### Yield

Yield is the estimation of wool remaining after the removal of contaminants in greasy wool. Contaminants may include vegetable matter, wax, suint, and dust. The price of wool is provided as a clean price (price on actual weight of wool). Therefore, yield is measured prior to the sale of raw wool.

### Vegetable matter

Vegetable matter refers not only to the type of cellulose contaminant in the wool but also to the amount present in the wool. Vegetable matter type and amount will not only affect the speed but also the degree of processing required. Both factors will, therefore, influence the level of price discounts received by producers. Additional carbonization may be required when there is a high vegetable matter percentage or when there is a high level of hardheads (plant seeds that have a hard seed coating). Carbonizing removes the vegetable matter by applying a known concentration of sulfuric acid to carbonize the cellulose material, which is then pulverized through a crushing and dedusting process (Teasdale, 1996). Vegetable matter, such as burrs, shive, and seed, can be removed in small volumes during carding and combing.

### Wool garment comfort, prickle, and moisture

Knitted woolen fabrics are more commonly used in casual wear, which is more popular and has superior comfort than the traditional woven wool fabrics. Knitted woolen fabrics were once worn as heavy outerwear, but now fine knitwear has been used in next-to-skin garments. Therefore, the pre-conceive association that wool can have prickle and an itch sensation requires attention. Garnsworthy et al. (1988) established that the physiological basis of the prickle sensation was the number of protruding fibers capable of exerting a force of ~0.74 mN against the skin. Wearer trials identified mean fiber diameter as the predominant measurement for prickle response (McGregor et al., 2013).

Prickle responses can also be influenced by skin moisture, such that moist skin evokes more neutral discharge from fabric, compared with dry skin (Garnsworthy et al., 1988). Single jersey wool fabrics showed that mean fiber diameter accounted for 53% and 56% of the variance in damp and sweaty sensations, respectively, with finer fibers associated with lower sensation scores (McGregor et al., 2015c).

### Wool ComfortMeter and Wool HandleMeter

Two instruments, the Wool ComfortMeter and HandleMeter, were developed to objectively measure the comfort and handle of woolen fabrics. These instruments were calibrated to wearer trials. The Wool ComfortMeter reading is strongly correlated with the prickle rating assigned by wearers (McGregor et al., 2013, 2015b; Naebe et al., 2015). The Wool HandleMeter measures the handle parameters of knitted single jersey wool fabric, using eight objective parameters to predict handle attributes (IWTO DTM-67, 2014).

## The Future of Animal Fiber Production: Sustainability and Skin Health

**Figure 4.**
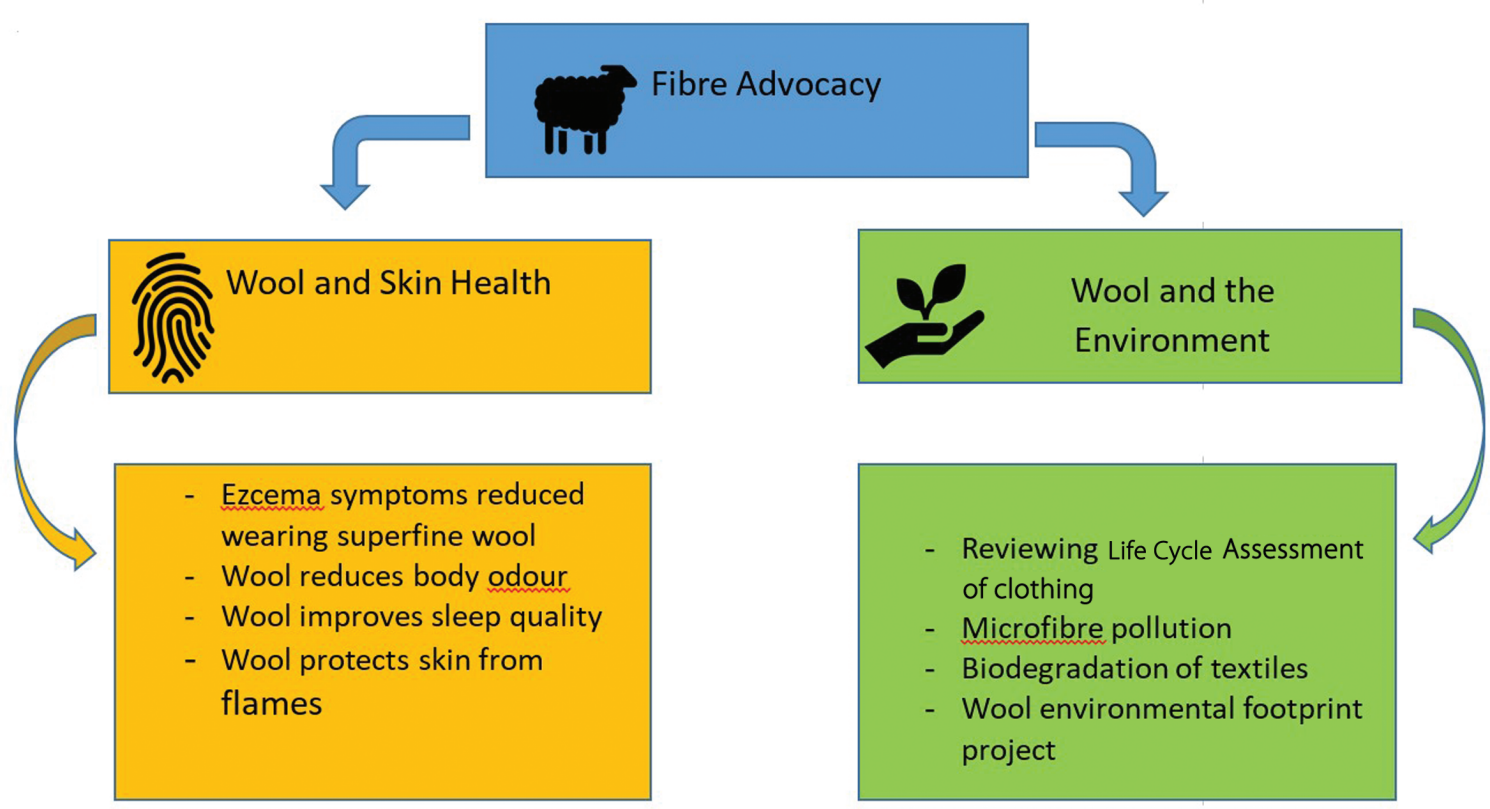
An overview of fiber advocacy for current wool research.

The attributes of wool to improve skin health and environmental sustainability profile of wool are two areas of considerable interest to research funding bodies (Figure 4). Recent work has seen the opportunities for wool to further promote itself with these credentials. The following chapter will highlight some of the recent work.

### Microparticles and pollution

Fiber content of textiles is often not the source of consumer attraction, particularly in fast fashion. Consumers do not consider or are aware that they are wearing “plastic,” when purchasing polyester garments, and textile engineering often manufactures synthetics to feel like organic fibers at a reduced cost. However, polyester fibers will pill, break, and wear down creating the same pollution issues as plastic bags.

Currently, two-thirds of all textile items are synthetic, petroleum, based polymers, and consideration is being made on including a metric for microplastic pollution in the environmental assessment of textile sustainability (Henry et al., 2019). The emerging issue of microfiber pollution in our waterways and the impact on the ecosystem puts natural fibers in the spotlight as a more sustainable fiber for apparel use.

Microplastics pollution caused by washing synthetic textiles is one of the main sources of pollution found in oceans, waterways, on land, and in the air. Synthetic clothes contribute to about 35% of global microplastics in the world oceans. A study investigating the washing process of synthetic clothing showed that between 124 and 308 mg of microfibers for each kilogram of washed fabric is filtered from wastewater. The length and diameter of the microfibers indicate dimensions that would pass through wastewater treatment and pose a threat to marine organisms (De Falco et al., 2019). The annual microfiber pollution from apparel into the marine environment is estimated at 0.2 million tonnes annually (Sherrington, 2016).

The textile industry is not certain of the environmental impact of these synthetic fibers, but the recent analysis of biodegradation in seawater was measured using the percentage of material converted to carbon dioxide over a 90-d test period. The synthetic fibers, polyester, nylon, and polypropylene were compared with Merino wool knit fleece and wool carpet pile. Commercial wool products degraded between 20% and 23% in 3 mo, an even greater degradation than the cellulose control material (10% degradation). The synthetic products had either no degradation (polypropylene) or up to 1%. The authors concluded that wool fibers readily biodegrade in seawater and would only persist for a period of months, compared with synthetic fibers existing for many years or decades (Collie et al., 2019). A concerning report of microfibers in the Hudson River and entering the Atlantic ocean showed that 50% of microfibers are plastic in origin (Miller et al., 2017).

### Skin health

Individuals with sensitive skin or atopic dermatitis would not naturally recognize wool as a fiber choice to wear next to skin. However, sensitive skin loses the ability to regulate moisture, and wool has the highest moisture-absorbing capacity of all apparel fibers. Wool fiber has the ability to transfer moisture between the body and the environment, which achieves thermal comfort and a stable microclimate between the skin and fabric (Li et al., 1992). A recent study evaluated the effects of quality of life and physiological measures of adults and children with atopic dermatitis while wearing 17.5 µm fine Merino wool for 6 wk compared with standard clothing for 6 wk. The Merino wool clothing provided improvements in mean eczema area and severity index scores and dermatology life quality index scores, compared with standard clothing (Fowler et al., 2019).

The use of superfine wool has also been examined in infantile eczema in patients between 4 wk and 3 yr of age. The crossover study compared 100% superfine wool clothing with 100% cotton clothing, with each fiber being worn for a period of 6 wk. The superfine wool clothing reduced SCORing Atopic Dermatitis index, severity index, Infants Dermatitis quality of life index, and topical steroid use, while changing from wool to cotton resulted in an increase of these scores. The conclusions made by the authors suggested that superfine Merino wool can be used to manage childhood atopic dermatitis (Su et al., 2017).

The use of wool to improve other health conditions has also been investigated using woolen underwear, bed coverings, mattresses, and woolen cushions. Patients with fibromyalgia, which is a debilitating disease causing chronic pain and tender points (Burkham and Haris, 2005), require a multidisciplinary approach to treatment that may include the use of wool. Patients using wool for a period of 20 wk showed significant improvements in pain score, tender points count, and sleep quality index, compared with a baseline assessment of 7 wk without wool products (Kiyak et al., 2009).

## Conclusions

The following review has provided an overview of the level of scientific understanding that is incorporated into sheep and wool production. In terms of global market share, wool is considered a small niche product, but it has successfully positioned itself as a superior product in terms of its eco credentials and advantages to human health and well-being. Like all agricultural products, wool continues to aim to achieve greater efficiencies in production and quality. These efficiencies may stem from improved on-farm management, continued genetic improvement in wool production, and quality or reduced cost of production through innovation and technology. This approach will continue to maintain the competitiveness of the wool fiber.

## About the Authors

**Emma Doyle** completed her PhD in sheep parasitology and genetic selection. Emma is a senior lecturer and researcher at the University of New England and has been teaching for the past 14 yr, specialized sheep and wool units offered by distance education for students across Australia. Her research is in a number of areas: small ruminant parasitology, vaccination against internal parasites and immune-stimulants, drench efficacy, sheep reproduction, guardian animals, wool fabric comfort, shorter shearing intervals, and social factors affecting the sheep and wool industry.

**James Preston** holds a degree in Animal Science and Management from The University of Melbourne and completed a PhD in Fibre Science from the Institute of Frontier Materials at Deakin University. Previously, James has held roles within the NSW Department of Primary Industries working on topics associated with the importance of wool quality in the Australian wool industry. He currently works at The University of New England as a sheep and wool Lecturer.

**Bruce McGregor** holds degrees of Agricultural Science Honours (The University of Melbourne) and PhD in Fibre Science and Textile Technology (University of New South Wales). As an animal nutrition scientist, he conducted long-term studies with Angora and cashmere goats, Merino sheep, and alpacas on the nutritional requirements for fiber growth and factors affecting animal fiber quality and skin follicle development. His textile research evaluated the performance of cashmere and wool knitwear and factors affecting cashmere dehairing efficiency. In a national collaborative program, he investigated objective measurement and consumer comfort assessment of wool and cashmere next-to-skin knitwear. He has also undertaken textile value chain assessments in developing countries.

**Phil Hynd** is the Emeritus Professor in Animal and Veterinary Sciences at The University of Adelaide. After a career focused on research in animal nutrition, wool growth and animal physiology, and teaching in agriculture, animal sciences, and veterinary sciences, he is now dedicated to other activities supporting the animal industries. He is currently the Chair of the Expert Panel for Meat and Livestock Australia, a member of The Animal Welfare Collaborative Advisory Committee, a private consultant, and also involved in ongoing research in wool harvesting and fetal programming. He is the President of the SA branch of the Australian Association of Animal Sciences.
